# Linking leaf traits to growth responses under climate warming in tropical trees

**DOI:** 10.3389/fpls.2025.1721483

**Published:** 2025-12-02

**Authors:** Anna Gardner, Sebastián González-Caro, Mirindi Eric Dusenge, Zorayda Restrepo Correa, Iain P. Hartley, Patrick Meir, Lina M. Mercado

**Affiliations:** 1 Faculty of Environment Science and Economy, University of Exeter, Exeter, Devon, United Kingdom; 2 School of Biological Sciences, University of Birmingham, Birmingham, United Kingdom; 3 Instituto de Biología, Universidad de Antioquia, Medellín, Colombia; 4 Division of Plant Sciences, Research School of Biology, The Australian National University, Canberra, ACT, Australia; 5 Grupo de Servicios Ecosistémicos y Cambio Climático, Corporación COL-TREE, Medellín, Colombia; 6 School of Geosciences, University of Edinburgh, Edinburgh, United Kingdom; 7 UK Centre for Ecology and Hydrology, Crowmarsh-Gifford, Wallingford, United Kingdom

**Keywords:** climate warming, plant functional traits, acclimation, tropical forests, montane forest

## Abstract

Climate warming is affecting the composition and distribution of Andean tropical montane forests (TMFs), resulting in varied growth responses among tree species. However, the underlying mechanisms driving these growth responses to climate warming remain largely unexplored. To address this knowledge gap, we investigated the role of leaf functional traits in mediating growth responses to temperature using a common garden experiment across a 2000 m thermosequence in the Colombian Andes. Fifteen dominant Andean tree species—originating from lowland and montane habitats—were grown under common soil and water conditions at three elevations. This experimental design exposed montane and lowland species to their native thermal environments as well as to warming and cooling respectively, thereby simulating upward migration consistent with documented shifts along elevation gradients. We measured 19 traits related to photosynthesis and its temperature response, thermotolerance, leaf structure and water use and assessed their associations with relative growth rates under warming and cooling conditions. Trait-growth relationships varied with thermal context. Thermal tolerance, photosynthesis and its temperature response, water use and leaf structural traits were consistently linked to growth explaining 88-91% of its variability across most thermal environments. However, for montane species under warming conditions, only thermal tolerance and photosynthetic traits remained significant, explaining 48% of the observed growth variability. Thermal acclimation of traits played an important role in mediating growth responses to temperature change. Traits associated with growth did not differ between species groups under native conditions but diverged under altered thermal environments. Collectively, these findings suggest that traits related to alternative physiological processes become increasingly relevant for montane tree species under climate warming. Our findings demonstrate that climate warming alters trait-growth relationships and highlights key functional traits that mediate growth responses to temperature.

## Introduction

Tropical montane forests (TMFs) consist of just 8% of tropical forest area (Spracklen and Righelato, 2014), yet they are vital biodiversity hotspots (Myers et al., 2000) and significant carbon sinks (Cuni-Sanchez et al., 2021; Duque et al., 2021). Despite their ecological importance, TMFs are particularly vulnerable to future warming due to the narrow thermal tolerances of their constituent species, the limited availability of higher-elevation habitat, and the high physiological sensitivity of montane trees to temperature increases (Booth et al., 2012; Friedlingstein et al., 2014). Many tropical species have evolved under stable thermal conditions, resulting in narrow thermal tolerances and thus narrow elevational ranges (Janzen, 1967; Perez et al., 2016). Indeed, studies have demonstrated that some tropical tree species are already operating at temperatures beyond their photosynthetic (Dusenge et al., 2021) and germination (Sentinella et al., 2020) thermal optima, making them particularly vulnerable to rising temperatures (Coumou and Robinson, 2013). For example, by 2100, maximum daily temperatures in tropical montane regions may increase by up to 5 °C (Pabón-Caicedo et al., 2020). These changes threaten high-elevation specialists, potentially reducing productivity (Doughty and Goulden, 2009; Sullivan et al., 2020) and driving longer-term shifts in community composition (Duque et al., 2015; Fadrique et al., 2018; Feeley et al., 2011).

These compositional shifts – termed ‘thermophilisation’ – are characterised by the increasing abundance of warm-affiliated, lowland species (LS) as they migrate into cooler, high-elevation areas (Duque et al., 2015; Feeley et al., 2011). Thermophilisation has been documented across the tropical Andes, Central America, and Afromontane forests (Cuni-Sanchez et al., 2024; Fadrique et al., 2018; Tanner et al., 2022) and appears to be driven by two concurrent processes: the increased mortality of montane species (MS) at their lower, warmer range limits and by upward migration of LS into higher elevations (Duque et al., 2015; Fadrique et al., 2018). Under projected warming scenarios, MS (typically cold-affiliated) may suffer higher mortality due to physiological stress. In contrast, LS may successfully establish at higher elevations if their traits remain functional under cooler temperatures. Additionally, increasing competition from LS may further disadvantage MS. Despite these observed shifts, the physiological mechanisms underlying these compositional shifts remain unclear (Bahar et al., 2017; van de Weg et al., 2012). Although field-based physiological measurements in tropical systems are challenging, studying plant functional traits, such as morphological, physiological, and biochemical characteristics, offers a plausible approach to understanding the dominant mechanisms that influence species performance and, ultimately, ecosystem functioning.

Functional traits offer a powerful framework for predicting species’ responses to environmental change and are increasingly used to inform global vegetation models (Díaz et al., 2016; Kunstler et al., 2016). Numerous studies have examined how traits relate to climate–productivity relationships in forests worldwide (Harper et al., 2016; Šímová et al., 2019; Liu et al., 2021). However, the ability of traits to predict growth responses in a changing climate remains uncertain (Bauman et al., 2022; Rowland et al., 2021). While some studies report strong links between certain traits and performance (Kretz et al., 2025; Medeiros et al., 2019; Rowland et al., 2021), others find weak or inconsistent patterns (Paine et al., 2015; Wright et al., 2004). These inconsistencies may arise from the use of species-level means (Falster et al., 2018), or from variation in trait–growth relationships across spatial or environmental gradients (Messier et al., 2017). The relationship of leaf-level photosynthetic traits and plant growth has been widely studied due to its mechanistic foundation, that higher photosynthetic rate should, other factors remaining approximately constant, lead to faster growth (Shipley et al., 2006). Nevertheless, the evidence remains mixed (Gibert et al., 2016; Poorter et al., 2009). In water-limited environments, hydraulic traits often predict growth better than photosynthetic traits (Kretz et al., 2025; Rowland et al., 2021), highlighting the importance of water relations for plant survival and suggesting that trait-growth relationships could be context-dependent, especially in relation to limiting environmental conditions. In mesic environments, as temperatures rise, thermal conditions may also impose physiological constraints. This raises the question of whether thermal traits become increasingly relevant when temperatures deviate from species’ optimal ranges, as species experience cooling stress during upward elevational migration or warming stress at their current elevations.

Leaf traits associated with photosynthetic thermal responses and thermotolerance may provide valuable insights into species’ growth responses to rising temperatures and thermal stress (Perez et al., 2020; Tarvainen et al., 2022), particularly in tropical montane species currently experiencing such conditions. For instance, plants operating above their photosynthetic thermal optimum (T_optA_) may reduce carbon uptake (Slot and Winter, 2016; Varhammar et al., 2015), ultimately limiting growth (Reich et al., 2022). However, some species have been found to tolerate supra-optimal temperatures and continue growing (Dusenge et al., 2023; Restrepo et al., 2025), suggesting different strategies for coping with heat stress. If montane plants can acclimate their photosynthetic temperature response to increased warming, they may be able to maintain growth under future climate (Dusenge et al., 2025; Restrepo et al., 2025). Additionally, strategies related to thermal regulation, such as increased transpiration via thermoregulatory traits such as high stomatal conductance or water-use efficiency (WUE), and variation in morphological traits such as increased leaf area, may also contribute to heat tolerance (Fauset et al., 2018; Perez and Feeley, 2020). Furthermore, increased tolerance to high temperature, as indicated by thermotolerance traits such as T_50_ (the temperature at which PSII function declines by 50%), could be key indicators of resilience (Tarvainen et al., 2022; Valliere et al., 2023). Together, these strategies suggest that montane plant species are likely to adjust multiple traits to cope with increased warming.

Lowland species expanding into cooler, high-elevation environments may also modify structural and nutrient-related traits (e.g. leaf nitrogen, phosphorus, or thickness) to maintain physiological function under suboptimal temperatures (Crous et al., 2022; Dusenge et al., 2020; Poorter et al., 2009; Sage and Kubien, 2007). Specifically, plasticity of leaf thermal traits for lowland species growing in cooler-than-native thermal environments has been reported with structural and water use traits and leaf nitrogen content becoming more conservative than under native thermal conditions (Cox et al., 2024), indicating that trait–growth relationships may differ systematically between MS and LS, depending on their thermal context. Therefore, detailed measurements of key traits involved in photosynthesis, thermoregulation and thermotolerance are expected to significantly enhance our understanding of the mechanisms influencing tree growth, particularly in the context of climate warming (Ntirugulirwa et al., 2023; Tarvainen et al., 2022). Empirical data linking these traits to plant growth responses under warming, especially in species-rich tropical forests, remains scarce (Doughty and Goulden, 2009; Verryckt et al., 2022). Measuring such traits in the field is resource-intensive, however common garden experiments combined with natural elevation gradients offer a valuable approach for exploring trait–growth dynamics under more controlled, yet ecologically realistic, warming and cooling scenarios (Schwinning et al., 2022; Silveira et al., 2019).

In this study, we used a common garden experiment along a natural temperature gradient (thermosequence) in the Colombian Andes to examine growth responses to temperature change in 15 dominant tropical tree species. We focused on both MS and LS exposed to temperatures within and outside their native thermal ranges (i.e., warming for MS and cooling for LS) to simulate the effects of thermophilisation. We collected data on 19 functional leaf traits related to photosynthesis, thermal responses, thermotolerance, water use, structure, and chemistry, with the aim understanding their relationship with relative growth rates (RGR) in juvenile trees. Specifically, we addressed the following questions: a) Which functional leaf traits are strongly associated to juvenile tree growth under native and non-native thermal environments? And b) Do montane and lowland species differ in how traits mediate their growth responses across temperature treatments?

We expected photosynthetic traits to be associated with tree growth, particularly in native environments where conditions are favourable. Moreover, given the increasing evidence that structural and thermal physiological traits shape species’ performance under thermal stress (Tarvainen et al., 2022; Valliere et al., 2023), we hypothesised that leaf traits related to structure (e.g. leaf mass per area, LMA) and thermotolerance (e.g. the temperature at which the maximum quantum yield of PSII (F_v_/F_m_) declines by 50%, T_50_) would be strongly associated with growth in non-native thermal environments. Specifically, we expected that montane species (MS), when exposed to warming, would exhibit stronger growth associations with structural traits, reflecting the adaptive value of robust leaf construction under high-temperature stress (Poorter et al., 2009). Conversely, we anticipated that lowland species (LS), when transplanted to cooler, high-elevation environments, would show growth associations with both structural and thermotolerance traits, reflecting the dual challenge of maintaining leaf function and photosynthetic performance under suboptimal temperatures (Crous et al., 2022; Dusenge et al., 2020; Sage and Kubien, 2007). Finally, we expect that both thermal acclimation of physiologically related traits and thermal plasticity of other traits (e.g. structural) play roles in mediating growth responses under changing thermal environments.

## Materials and methods

### Experimental sites

This study was conducted in the north-western region of the Colombian Andes across three common garden tree plantations located along a natural elevation gradient (Restrepo et al., 2025). These sites allowed us to expose tropical montane (MS) and lowland (LS) species to temperatures close to the centre of their observed thermal distribution (i.e. “native” temperatures), as well as to temperatures beyond their native range (i.e. warming or cooling). This design mimics thermophilisation trends observed in Andean forests (Duque et al., 2015; Fadrique et al., 2018), enabling an assessment of species’ responses to thermal shifts.

The three experimental sites span a ~2000 m elevation gradient and are located within ~40 km of each other (straight-line distance). Each site represents a distinct temperature regime within the species’ thermal niches and is named after its mean annual temperature, i.e. ‘14°C site’, ‘22°C site’ and ‘26°C site’ (**Table 1**). All sites employed a common garden approach. Trees were planted in open space using a standard soil medium (400 kg per tree) collected near the 14°C site, chosen for its similarity in chemical composition to the surrounding native forest soils. This soil was transported to all three sites to minimise the influence of soil variation on plant performance. Although all sites receive over 2000 mm of annual precipitation, irrigation was applied after two consecutive rain-free days to prevent water limitation. On average, 8–24 litres of water per tree per night were used during dry periods. Further information about experimental sites and design, study species, soil nutrients and climate are available in Restrepo et al. (2025).

**Table 1:** Climate at experimental sites.

Site characteristics	14°C site	22°C site	26°C site
Latitude Longitude	5.513°N −75.678°W	5.541°N −75.685W	6.844°N −75.810°W
Elevation (masl)	2516	1357	736
MAT (°C)	13.78	22.14	25.58
*T*_day_ (°C)	16.1	22.5	27.1
*T*_night_ (°C)	12.6	19.6	22.7
MAT (10%; °C)	11.6	18.3	20.7
MAT (90%; °C)	18.4	26.4	32.3
MAP (mm yr^−1^)	2774	2045	2298
VPD_day_ (kPa)	0.82	1.14	1.83
VPD (90%; kPa)	1.57	2.24	3.17
Direct PAR mean (5% - 95%; μmol m^-2^ s^-1^)	580 (17-1507)	682 (4-1864)	NA
Diffuse PAR mean (5% -95%; μmol m^-2^ s^-1^)	368 (20-809)	332 (3-768)	NA
Maximum number of consecutive days without rain	12.5	21.4	20.3

Information taken from Restrepo et al. (2025) corresponding to weather station data for the period October 2019 -January 2022. Mean daytime (*T*_day_) and night-time (*T*_night_) temperatures were calculated for 06:00–17:59 and 18:00–05:59 respectively. Mean annual temperature (MAT) and mean annual precipitation (MAP) include the 1% and 99% percentiles. Daytime vapour pressure deficit (VPD_day_) was calculated using values over 06:00–17:59.

### Seed collection and propagation

Before planting, seeds were collected for all species from a single montane rainforest site located near the 14°C experimental plantation (1300–2500 masl), to minimise intra-specific variation driven by local environmental adaptation. Seeds of montane species were collected from higher elevations (2200–2500 masl), where ambient temperatures are close to their growth optimum. Seeds of lowland species were collected from lower elevations (1300–2200 masl), representing the coolest limit of their natural thermal range. For each species, seeds were collected from 3 to 5 trees to further reduce intra-specific genetic variation. Seeds were propagated in polypots in a nursery near the 22°C site for 8–24 months, depending on species-specific germination rates. At the time of planting (November to December 2018), saplings ranged from 50 to 100 cm in height.

### Experimental design

At each experimental site, individuals of 15 tree species—selected from the 27 most dominant species in Colombian tropical montane forests (TMFs; **Table 2**)—were planted in four 600 m² plots. Each plot was divided into six 94 m² blocks, with one individual of each species planted in each block (15 species × 6 blocks × 4 plots = 360 trees per site). Trees were spaced 2.5 m apart to minimise competition and were randomly assigned to planting locations. One block per plot received nutrient fertiliser every four months to reduce soil nutrient limitations. However, for the purposes of this study, we analysed only trees grown in the five unfertilised blocks per plot. These trees were planted in nutrient-rich soil sufficient to support healthy growth without supplementation. The 15 species used in this study were categorised as either MS or LS based on their thermal ranges Restrepo et al. (2025) (**Supplementary Table 1**). Of these, 12 species (eight MS, four LS) were included in the final analysis.

**Table 2:** Thermal distribution of study species: mean temperature (T Mean) (°C), minimum temperature (T Min) (°C) and maximum temperature (T Max) (°C) of species’ thermal ranges.

Species	T mean (°C)	T min (°C)	T max (°C)
Lowland			
*Inga marginata*	22.7	15.2	30.4
*Inga ingoides*	25.4	17.1	32.0
*Inga* sp*ectabilis*	25.4	19.8	30.9
*Inga densiflora*	23.1	17.4	29.2
Montane			
*Clethra fagifolia*	15.3	9.9	20.3
*Quercus humboldtii*	16.4	11.8	21.2
*Clusia multiflora*	13.4	7.1	19.1
*Guatteria lehmannii*	16.6	11.4	21.5
*Ilex laurina*	16.4	11.6	22.1
*Miconia theizans*	17.2	9.9	24.0
*Weinmannia pubescens*	15.0	9.3	19.9
*Clusia ducu*	12.9	6.7	18.7
*Chrysochlamys colombiana*	17.3	12.2	22.2
*Hieronyma antioquensis*	15.2	10.7	19.7
*Andesanthus lepidotus*	13.2	7.2	19.1

Information taken from Restrepo et al., 2025.

### Thermal treatments

Species were assigned to site-specific thermal treatments based on their native temperature distributions (**Table 3**). The 14°C site, which corresponds to the centre of their native thermal distribution for MS species, was treated as the control for montane species (“MS Control”), while the 22°C site represented a warming treatment (“MS Warming”). For LS species, the 22°C and 26°C sites were within their native thermal optima and thus considered control sites (“LS Control”), while the cooler 14 °C site represented a cooling treatment (“LS Cooling”).

**Table 3:** Species groups and site and temperature treatment categorisation.

Category name	Abbreviation	Site MAT (°C)	Species affiliation	Number of species
Montane Species Control	MS Control	14	Montane species	11
Montane Species Warming	MS Warming	22	Montane species	11
Lowland Species Control	LS Control	2 species at 22 and 2 species at 26	Lowland species	4
Lowland Species Cooling	LS Cooling	2 species growing at 14 and 2 species growing at 22	Lowland species	4

### Tree growth and functional trait sampling

Stem diameter was measured quarterly from January 2019 until February 2022 for all 15 tree species at each experimental site (Restrepo et al., 2025). To increase measurement precision and detect short-term growth changes, all trees were marked ~2 cm above the soil surface to avoid irregularities at the base. Relative growth rate (RGR) was calculated from stem diameter (D) using the log-difference method:

(1) $$RGR=[log(D_{i})-log(D_{0})]/(t_{i}=t_{0})$$

With *D* is expressed in millimetres (mm), (*t*_i_ – *t*_0_) in years and RGR in mm mm^-1^ year^-1^.

### Leaf trait measurements

A leaf trait measurement campaign was conducted in January 2022 across all experimental sites, during which 19 leaf functional traits were assessed (see **Table 4** for full details, trait acronyms, descriptions, and ranges). These traits were selected to represent key physiological, thermal, and structural properties of the trees and were grouped into five functional categories. Photosynthetic traits included the maximum carboxylation capacity (V_cmax_), the maximum electron transport capacity (J_max_), and the net photosynthetic assimilation rate (A_net_) at saturating irradiance (see **Table 4**), leaf nitrogen content (N) and phosphorus content (P). Temperature-response traits encompassed the thermal optima for V_cmax_ (T_optV_), J_max_ (T_optJ_), and A_net_ (T_optA_); the activation energy for V_cmax_ (E_aV_) and J_max_ (E_aJ_); the V_cmax_ and J_max_ values at their respective thermal optima (V_cmaxOpt_ and J_maxOpt_); and the net photosynthesis rate at the thermal optimum (A_opt_) under saturated irradiance. Thermotolerance was assessed via two traits: T_max_, the high-temperature CO_2_ compensation point, and T_50_, the temperature at which the maximum quantum yield of PSII (F_v_/F_m_) declines by 50%. Water-use traits included stomatal conductance (g_s_) at saturating irradiance and carbon isotope discrimination (δ¹³C). Lastly, structural traits included leaf mass per area (LMA) and leaf carbon content (C). Photosynthetic, structural, water use and chemical trait measurement protocols are fully described in Cox et al. (2023 and 2024) and Dusenge et al. (2025) and are available in the supplementary (**Supplementary Methods 1**). Time-intensive photosynthetic and temperature response traits including *g*_s_ and T_max_ were measured from one leaf per species per plot per site (n = 4 in most cases, and n = 3 for some species), and the same leaves were subsequently harvested for chemical analysis. Structural traits were sampled more intensively, with 12 leaves per species per site, typically collected as one leaf per tree from three blocks within each plot.

**Table 4:** List of all measured traits, descriptions, ranges of values, standard error values and sample sizes for montane (MS) and lowland (LS) species groups in either the Control (C) or temperature change conditions (i.e. warming or cooling).

Trait	Description	Range of trait values measured in montane species (MS)		Range of trait values measured in lowland species (LS)	
		MS control	MS warming	LS control	LS cooling
V_cmax_	Maximum carboxylation capacity (µmol CO_2_ m^-2^ s^-1^) at the reference temperature (25°C) at saturating photosynthetic photon flux density (PPFD) (1800 µmol m^-2^ s^-1^)	19.9-126.6	10.1-103.7	31.8-103.2	9.9-91.2
J_max_	Max. electron transport capacity (µmol CO_2_ m^-2^ s^-1^) at the reference temperature (25°C) at saturating PPFD (1800 µmol m^-2^ s^-1^)	42.1-259.1	16.4-166.6	44.8-167.8	36.5-125.1
A_net_	Net photosynthetic assimilations (µmol CO_2_ m^-2^ s^-1^) at the reference temperature (25°C) at saturating PPFD (1800 µmol m^-2^ s^-1^)	1.6-22.6	2.3-19.9	7.8-23.7	1.27-18.7
*g*_s_	Stomatal conductance (mol H_2_O m^-2^ s^-1^) at the reference temperature (25°C) at saturating PPFD (1800 µmol m^-2^ s^-1^).	0.02-0.26	0.01-0.27	0.1-0.39	0.04-0.24
T_optV_	Thermal optimum of V_cmax_ (°C)	21.7-36.8	27.5-36.5	29.5-35.2	27.7-34
T_optJ_	Thermal optimum of J_max_ (°C)	21-34	25.4-35.5	26.7-37.6	27.6-34.4
Ea*_V_*	Activation energy of V_cmax_ (KJ mol^-1^)	31.3-147.4	32.9-163.9	28.8-113.5	28.7-95.8
Ea*_J_*	Activation energy of J_max_ (KJ mol^-1^)	14.3-133.4	26.3-101.3	32-109	32-75.4
V_cmaxOpt_	V_cmax_ at the optimum temperature (µmol CO_2_ m^-2^ s^-1^)	33-270.9	13.65-159.4	36.4-189.8	18.3-146.4
J_maxOpt_	J_max_ at the optimum temperature (µmol CO_2_ m^-2^ s^-1^)	34.1-45.8	19.2-203.9	52.7-231.3	47.8-171.5
T_optA_	The thermal optimum of net photosynthesis from measured temperature response of A_net_.	14.12-27.4	22.2-29.4	19.2-29.2	16.9-30.3
A_opt_	Net photosynthesis rate at the thermal optimum estimated from measured temperature response of A_net_ (i.e., at measured intercellular CO_2_ concentration C_i_.) (µmol CO_2_ m^-2^ s^-1^)	3.4-21.7	2.2-20.5	7.4-23.3	1.8-19.5
T_max_	The upper temperature at which net CO_2_ assimilation rates are zero (°C)	34.1-45.8	36.5-47.5	39.3-44.4	39.4-41.3
T_50_	The upper temperature at which net CO_2_ assimilation rates are 50% (°C)	37.8-40.9	39.8-43.5	43.3-46.7	43.3-44.1
C	Leaf carbon content (g g^-1^)	38.2-46.2	36.7-46.5	40.9-46.5	42.2-47.3
N	Leaf nitrogen content (g g^-1^)	0.82-1.93	0.64-1.9	2.2-3.2	1.7-3.2
P	Leaf phosphorus content (g g^-1^)	550-1049	269-949	579-1530	301-1730
LMA	Leaf mass per area (g m^-2^)	86.1-247.4	28.5-504.8	48.7-150.6	85.3-228.2
*δ*^13^C	Ratio of ^13^C to ^12^C stable carbon compound (‰) in leaves	-29.8—25.3	-30—23.9	-32—28.7	-30.8—29.93

Additional details regarding the gas exchange methods used for the collection and parameterisation of physiological traits and chemical and structural traits are given in (Dusenge et al., 2025) and Cox et al. (2023) respectively.

T_50_ was estimated by evaluating the relationship between chlorophyll fluorescence and leaf temperature. Leaves were exposed to a range of temperatures (35, 37, 39, 41, 43, 45, 47, 49, 51, 53, 55°C), using an infrared lamp as heat source during 15 minutes, following the protocol described by Tarvainen et al. (2022). Leaf temperature was monitored with a handheld thermometer, and the lamp’s intensity was adjusted by varying its distance from leaf to reach the target temperature. Chlorophyll fluorescence was measured for each sample before the heat treatment and again 24 hours later, after dark storage, using an OS30p^+^ handheld fluorometer (Opti-Science). Fluorescence was used to assess PSII function, estimating maximum fluorescence after a saturating light pulse (F_m_), and the variable fluorescence (F_v_), calculated as F_v_/F_m_, which reflects the potential quantum efficiency of photosystem II. All F_v_/F_m_ values were standardized as relative to the pre-treatment measurement to quantify the reduction in PSII function. A logistic regression was performed between leaf temperature and standardized F_v_/F_m_ values using a nonlinear least squares model to estimate T_50_, defined as the temperature at which fluorescence declined to 50% of its initial value. We performed one temperature response of F_v_/F_m_ per species per site by sampling one leaf per tree, from three trees, in three blocks per plot for each species at each site (n = 3 × 3 × 4). Most leaves yielded enough material for duplicate sampling. Five replicate samples per temperature treatment were then randomly selected for analysis.

### Data analysis

We defined our metric of tree growth rate for further analyses as the 75% quartile relative tree growth rate (RGR_75_) (Alsayed et al., 2020; Schröder et al., 2005) per species per plot in each site (four data points per species) (Equation 1). We used RGR_75_ (the 75th percentile of relative growth rate) as it better represents the growth frontier of individuals within each site, reducing bias from zero or near-zero growth values that can arise from measurement error, localised stress, or herbivory. This approach minimises the influence of sampling uncertainties and site-specific anomalies, providing a more reliable link between functional traits and tree vigour (Schröder et al., 2005). We also calculated the mean value of all traits for individuals within each plot and site, obtaining four trait values per species per site, comparable to the RGR_75_ estimates used in further analyses.

To address our two main research questions, we applied multiple complementary statistical approaches. Two-way ANOVA was used to test the effects of temperature treatment and species group on trait and growth responses. Linear regression was applied to examine relationships between individual traits and growth. Structural equation modelling (SEM) was employed to explore multivariate trait interactions and their combined influence on growth. Finally, null model analyses were conducted to test whether observed trait–growth relationships differed from expectations under random assembly. Together, these approaches provide a comprehensive assessment of how traits mediate species responses to temperature across successional groups.

First, we evaluated differences in RGR_75_ and in measured leaf functional traits between species groups (LS and MS) and treatments (control vs warming or cooling, respectively) using two-way ANOVA. A significant difference across studied traits across sites was used to describe thermal plasticity (Cox et al., 2023, 2024; Dusenge et al., 2025). To evaluate possible changes in trait distribution and the range covered for each trait at each site, we tested for differences in trait variance between treatments within each species group using *F*-tests. We first assessed associations between individual leaf traits and juvenile tree growth, by performing linear regressions between each of the 19 plant functional traits (**Table 4**) and RGR_75_. We analysed independently each species group (MS or LS) and compared between control and temperature treatment (warming or cooling, respectively). Our analysis focused on climatically affiliated group-level responses (MS vs. LS) rather than individual species effects. While this approach captures broad functional patterns, it does not account for species identity, which we acknowledge as a limitation.

We then assessed whether a combination of traits influence tree growth and if these traits differ between species groups (i.e., MS vs. LS) and temperature treatments (i.e., control, warming, or cooling) using structural equation modelling (SEM; Grace et al., 2012). Separate SEMs were constructed for each combination of species group and temperature treatment: MS control, MS warming, LS control, and LS warming (**Table 1**), and a multi-group comparison approach was applied (Gana and Broc, 2019). Before conducting the SEM, we assessed covariation among the 19 leaf traits measured. We used the *rcorr* function in R to generate a Pearson correlation matrix with associated *p*-values. Pairs of traits with correlation coefficients above 0.7 were considered strongly correlated (Ratner, 2009). To reduce multicollinearity, we performed a principal component analysis (PCA) and selected the highest-loading trait on each of the first eight PCA axes. These selected traits were retained for inclusion in the SEM.

SEM was implemented using the *laavan* package in R (Lefcheck, 2016). Traits that remained for inclusion into the SEM per functional category were included. We constructed an initial mechanistic pathway model describing the relationships among functional traits and their collective influence on growth. We assumed that structural traits influence other trait categories.

Relationships among traits and between traits and RGR_75_ were all evaluated using a null model framework to facilitate analysis of individual trait effects. The null model assumed that: i) all traits influence RGR_75_, ii) species groups and temperature treatment could influence all traits and iii) all traits could influence each other. No prior assumptions were made about the direction (positive or negative) of these relationships. Trait effects were categorized as either direct (i.e., one variable influencing another) or indirect (i.e., co-varying without directional causality). To assess differences among species groups and treatments, we applied a multi-group SEM approach, partitioning data into four categories: MS control, MS warming, LS control, and LS warming. The same path structure was fitted to each subset, and differences in estimated path coefficients were used to determine whether trait-growth relationships varied among groups. All the variables in the SEM were scaled between 0 and 1 to enable comparisons of the magnitude and direction of standardized path coefficients. Model selection was based on the χ^2^ goodness-of-fit test and AIC, which quantified deviations from the null model.

## Results

### Variations in growth and leaf functional traits across thermal treatments and species groups

**Figure 1:**
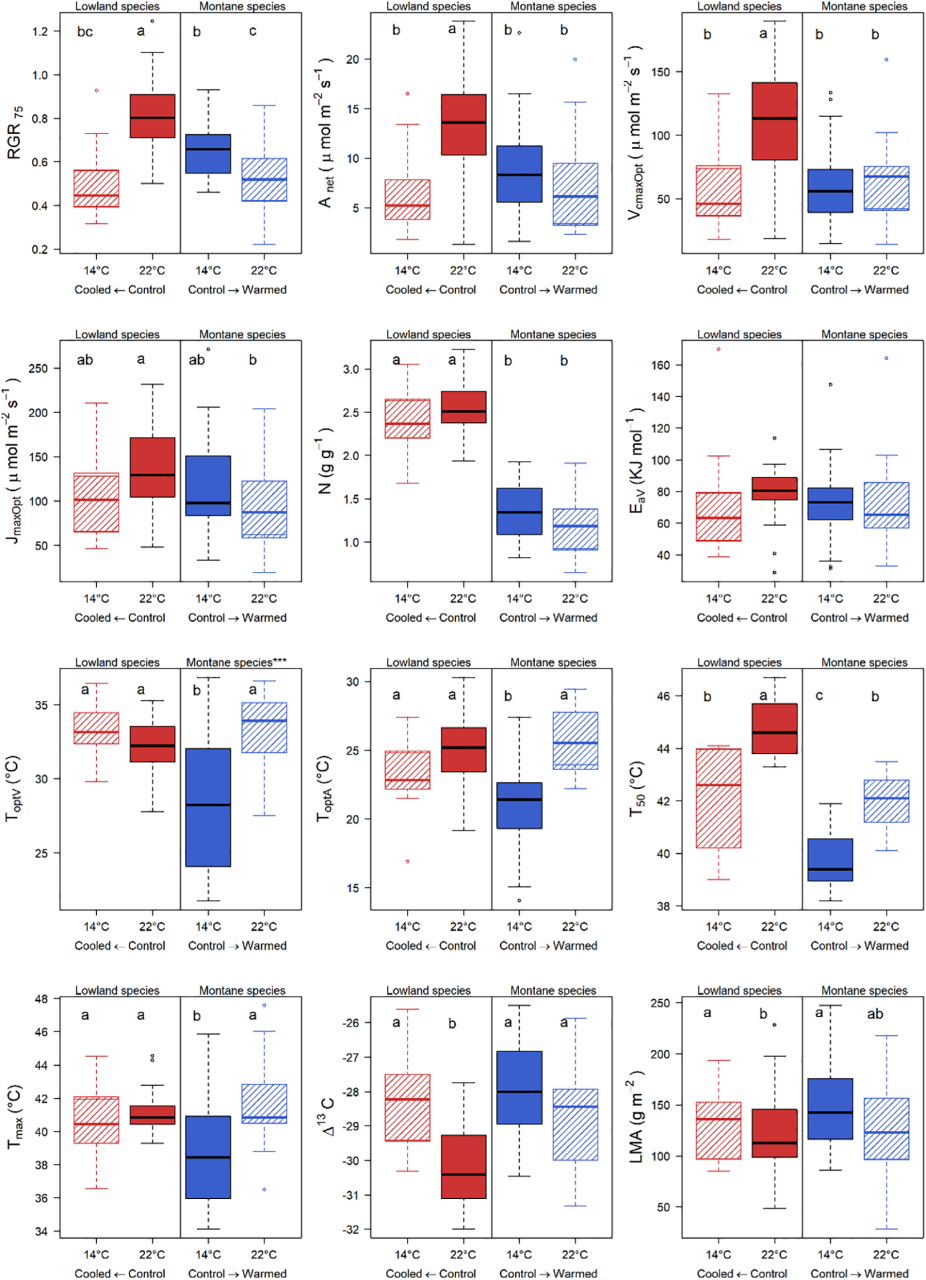
Variation in 75% relative growth rate (RGR_75_) and 11 leaf functional traits retained after the correlation-selection procedure. Red boxes represent lowland species, and blue boxes represent montane species. Filled boxes indicate control conditions, while striped boxes indicate treatments (cooling for LS and warming for MS). Letters denote statistically significant differences based on Tukey’s Honest Significant Difference test (*p* < 0.05). Asterisks above the boxes indicate significant differences in variance between treatments within the same species group, based on *F*-tests. Boxplots are shown only for functional traits used on SEM analysis. Lines separating LS and MS treatments are included only to help visualise comparisons of sites within each successional group. Statistical analyses (ANOVA) were performed across all groups.

We found strong differences in RGR_75_ and in several leaf functional traits across thermal treatments (**Figure 1**), the latter demonstrating thermal acclimation of physiological traits and thermal plasticity in remaining traits (e.g. structural traits). Growth in both MS and LS significantly declined when growing away from their native thermal environment, i.e. MS under warming and LS under cooling. Traits that significantly changed in MS with warming include temperature response traits (T_optA_, T_optV_) and thermotolerance (T_50_, T_max_) with remaining traits such as structural (LMA), water use (δ^13^C) and photosynthetic (N, V_cmaxOpt_, J_maxOpt_, E_aV_, A_net_) traits being unchanged. In LS under cooling, traits with significant change include photosynthetic (A_net_, V_cmaxOpt_), water use (δ^13^C) and structural (LMA) with remaining traits being unchanged.

### Which functional leaf traits are strongly associated to juvenile tree growth under native and non-native thermal environments?

**Figure 2:**
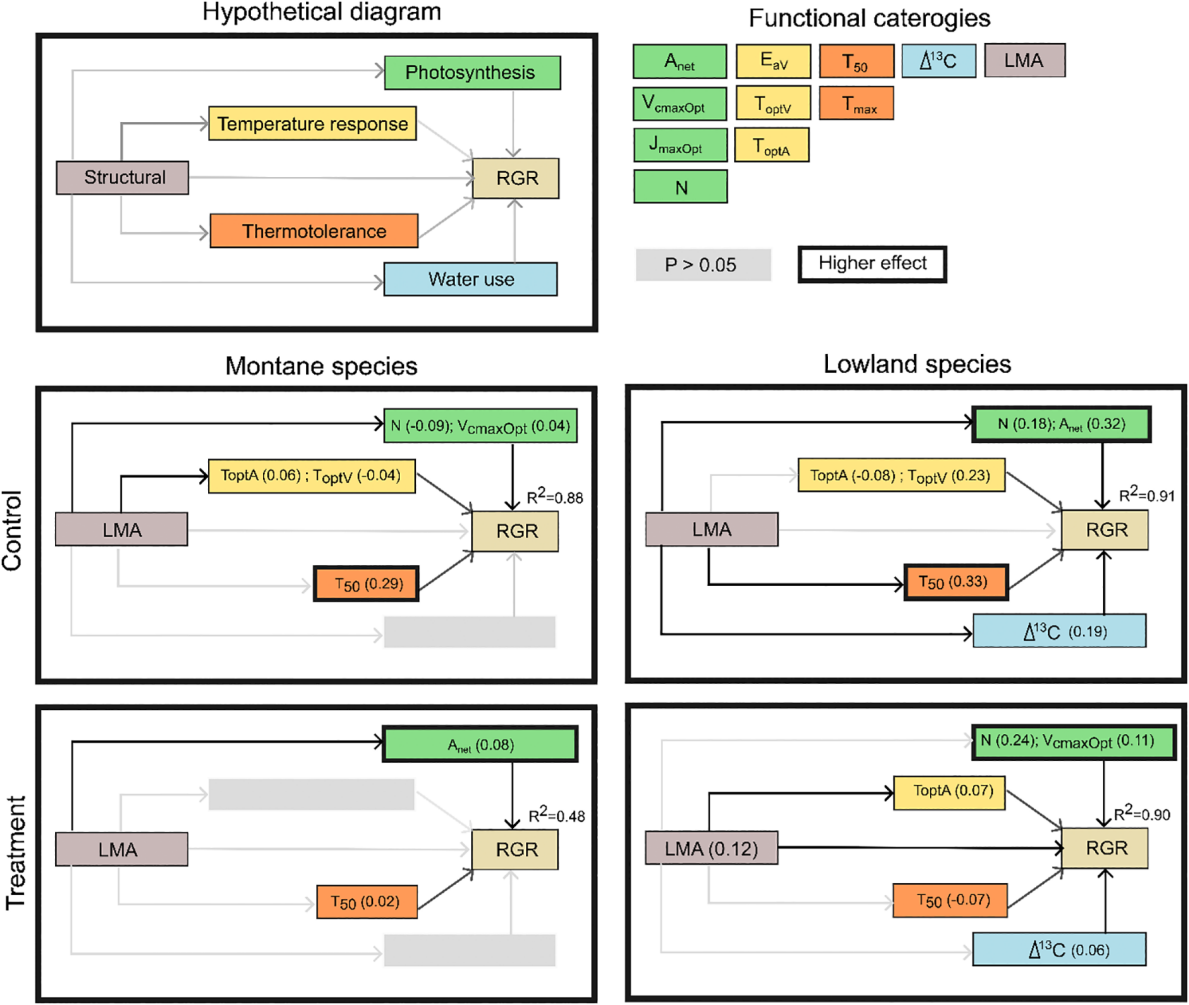
Results of the path analysis performed to examine the log-transformed mean 75% quartile growth rate (RGR_75_) under control and temperature condition plots for montane and lowland species. The hypothetical diagram illustrates the components used to classify the functional leaf traits, with colours indicating the functional categories. Black solid arrows represent significant relationships, while grey arrows indicate non-significant relationships. Standardised path coefficients are shown in brackets, and the coefficient of determination (R^2^) is provided for RGR_75_.

The linear regressions between each of the 19 studied plant functional traits with the 75% quartile relative growth rate (RGR_75_) reveal that nine traits (V_cmax_, J_max_, A_net_, g_s_, V_cmaxOpt_, J_maxOpt_, A_opt_, T_50_ and N) were significantly positively correlated with RGR in the montane species, at the native growth temperature (MS control, i.e., at 14°C) (**Table 5**). In this group, the trait with the strongest positive correlation with RGR_75_ was T_50_ (**Table 5**, *R*^2^ = 0.61, *p* < 0.001) followed by A_opt_ (*R*^2^ = 0.43, *p* < 0.001). For montane species growing at the extreme of their thermal ranges (MS warming, 22°C), only two traits (LMA and N) were significantly positively correlated with RGR_75_ (*R*^2^ = 0.27, *p* = 0.005 for LMA and *R*^2^ = 0.19, *p* = 0.019 for N, **Table 5**). In the lowland species growing at native growth temperature (LS control), three traits (T_optA_, T_50_, N) had a significantly positive correlation with RGR_75_. Here, the strongest positive correlation with RGR_75_ was obtained for leaf N (*R*^2^ = 0.5 *p* = 0.002) followed by T_optA_ (*R*^2^ = 0.32 *p* < 0.001). For lowland species growing under colder conditions (i.e. LS cooling), there were three traits (*T*_optA_, N, LMA) that were significantly correlated with RGR. Of these, LMA (*R*^2^ = 0.2, *p* = 0.026) had the strongest correlation with RGR_75_ (**Figure 2**, **Table 5**). A full correlation matrix of all traits is shown in **Table 5** and in **Supplementary Figure 1**.

**Table 5:** Results of bivariate linear regressions (correlation coefficient, R^2^) and p-value for each of the 19 leaf traits against relative growth rate at the 75% percentile (RGR_75_) for montane and lowland species in either control or with temperature change (warming or cooling).

Trait	Montane species				Lowland species			
	Control		Warming		Control		Cooling	
	*R* ^2^	*P*-value	*R* ^2^	*P*-value	*R* ^2^	*P*-value	*R* ^2^	*P*-value
V_cmax_	0.14	0.0037	0.03	0.41	<0.01	0.83	0.04	0.32
J_max_	0.3	0.001	0.03	0.39	<0.01	0.72	<0.01	0.96
A_net_	0.25	0.004	0.03	0.38	0.15	0.12	0.04	0.36
*g*_s_	0.28	0.002	0.03	0.37	0.18	0.09	0.03	0.39
T_optV_	0.03	0.33	0.05	0.28	0.08	0.27	0.03	0.38
T_optJ_	0.09	0.095	<0.01	0.89	0.12	0.17	0.02	0.518
E_aV_	<0.01	0.807	0.01	0.57	<0.01	0.83	<0.01	0.98
E_aJx_	0.10	0.085	0.03	0.38	<0.01	0.96	<0.01	0.66
V*_c_*_maxOpt_	0.26	0.003	<0.01	0.69	0.011	0.19	0.05	0.29
J_maxOpt_	0.41	<0.001	<0.01	0.89	0.05	0.4	<0.01	0.89
T_optA_	<0.01	0.79	<0.01	0.38	**0.32**	<0.001	0.16	0.029
A_opt_	**0.43**	<0.001	-0.037	0.91	0.036	0.22	<0.01	0.37
T_max_	0.09	0.095	0.01	0.609	0.01	0.67	0.14	0.072
T_50_	**0.61**	<0.001	<0.01	0.72	0.26	0.039	0.12	0.087
C	<0.01	0.67	0.11	0.092	0.09	0.12	<0.01	0.99
N	0.25	0.004	**0.19**	0.019	**0.50**	0.002	0.16	0.049
P	<0.01	0.67	0.11	0.092	0.04	0.44	0.02	0.47
LMA	0.01	0.54	**0.27**	0.005	0.03	0.506	**0.20**	0.026
*δ*^13^C	<0.01	0.76	0.05	0.25	0.06	0.35	0.03	0.42

Grey shaded cells indicate significant relationships at p < 0.05. The two highest significant R^2^ values in each of the groups are highlighted in bold as they are cited in the results section.

There were some commonalities in the obtained relationships across treatments (**Figure 2**). A_net_ is positively associated with RGR_75_ in all groups (*R*^2^ range of 0.03 to 0.25), although the relationship was only significant for MS under control conditions (*R*^2^ = 0.25, *p* = 0.04). LMA was significantly related to RGR_75_ only under temperature change treatments (i.e., MS warming and LS cooling), and the relationship was negative in lowland species under warming. Leaf N had a significant positive effect on RGR_75_ across all treatments, and T_50_ also significantly influenced tree growth under control conditions. At least one trait related to the temperature response of photosynthesis significantly influences RGR_75_ in all treatments, except for montane species under warming. Finally, neither of the water-use traits (i.e. *g_s_* and δ^13^C) was significantly related to tree growth in either species groups.

### Do montane and lowland species differ in traits that mediate their growth responses across temperature treatments?

We used structural equation modelling (SEM) to evaluate how measured leaf traits, along with species group and temperature treatment (control, warming, or cooling), influence juvenile tree growth, measured as relative growth rate (RGR_75_). Prior to building the SEM, a PCA was used to select the highest-loading trait on each of the first eight PCA axes to be retained for inclusion in the SEM. We retained eleven traits, each assigned to a specific functional category: photosynthetic traits (A_net_, V_cmaxOpt_, J_maxOpt_, N), temperature response (E_aV_, T_optA_ and T_optV_), thermotolerance (T_50_, T_max_), water use (δ^13^C), and structural (LMA). All reported path coefficients are standardised, allowing for direct comparison of effect sizes. Across all models, the SEMs performed very well (*p*-values or goodness of fit; **Figure 2**), with *R*² > 0.88 in three of the four models.

Overall, in all SEM models, at least one photosynthetic and one thermotolerance (T_50_) trait were retained. However, T_50_ showed a stronger influence on RGR_75_ in control than in temperature treatments. LMA had a direct effect on traits that are associated to growth in all treatments. Leaf nitrogen content (N) was significant in three treatments except for montane species under warming. δ^13^C was significantly associated to growth in both LS treatments (**Figure 2**).

Under control environments, a combination of photosynthetic temperature response traits, (photosynthesis and thermotolerance traits) were strongly associated to growth in both groups of species. Specifically, under MS control, five traits (N, V_cmaxOpt_, T_optA_, T_optV_ and T_50_) were retained in the final SEM, together explaining 88% of the variation in RGR_75_. Similarly, under LS control, N, A_net_, T_optA_, T_optV_, T_50_ and δ^13^C were retained, jointly explaining 91% of the variation in RGR_75_ (**Figure 2**).

Under altered thermal environments, in LS exposed to cooling, traits similar to those in the control environment were associated with growth: N, V_cmaxOpt_, T_optA_, T_50_, δ^13^C and LMA collectively accounted for 90% of the variation in growth. Under MS exposed to warming, only A_net_ and *T*_50_ were retained in the model, explaining 48% of the variation in RGR_75_ (**Figure 2**). When examining LS treatments alone (**Figure 2**), a similar set of traits explained 90-91% of the variability in RGR_75_ across both thermal environments, although the contribution of individual traits differed between treatments. Notably, under LS cooling conditions, the contribution of leaf nitrogen content increased while LMA showed a direct positive association with growth, whereas the remaining variables decreased in their relative contributions. In contrast, comparison of model outcomes for MS treatments revealed that under MS warming conditions, only two traits were directly associated with RGR_75_, collectively explaining just 48% of the observed variation. This contrasts markedly with MS control conditions, where five traits were directly associated with growth and explained 88% of RGR_75_1 variability.

## Discussion

This study explored how leaf traits mediate juvenile tree growth under climate warming in tropical montane forests (TMFs) of the Andes. Using a common garden experiment across a natural thermal gradient, we evaluated associations of key leaf traits and juvenile tree growth in montane (MS) and lowland (LS) species under both native and non-native temperature conditions. Our results provide empirical evidence showing that specific photosynthetic, temperature response of photosynthesis, structural, and thermotolerance traits significantly influence growth responses to changing temperature. These findings are critical for improving our understanding of species’ physiological responses to climate change and for refining trait-based models of forest productivity in tropical ecosystems.

### Trait associations to growth under native thermal conditions

Our findings demonstrate that leaf traits can explain a substantial portion of variation in juvenile tree growth under native thermal environments. Under control conditions, reflecting species’ native thermal environments, we found that photosynthetic traits were strongly associated with growth, consistent with previous studies (e.g. Rowland et al., 2021). Specifically, net photosynthesis at saturating irradiance (A_net_), optimal electron transport (J_maxOpt_), leaf nitrogen (N) and optimal temperature of Rubisco carboxylation (T_optV_), consistently explained a large proportion of growth variation in both montane (MS) and lowland (LS) species (*R*² = 88–91%). These results confirm the strong relationship between photosynthetic function and growth (Shipley et al., 2006) and support the need for inclusion of photosynthetic and temperature-sensitive physiological traits in predictive trait-based models. Despite being less commonly measured than morphological traits such as LMA (Dong et al., 2020; Kattge et al., 2019), photosynthetic traits demonstrated high predictive power, particularly for forests such as TMFs that are sensitive to temperature change and expected to experience rapid increases in temperature (Tovar et al., 2022; Pabón-Caicedo et al., 2020).

### Trait associations to growth under altered thermal environments

Most growth-associated traits varied with temperature in both montane and lowland species groups, which may reflect thermal plasticity and/or underlying genetic differences among individuals, suggesting that these factors together play important roles in mediating growth responses to changing thermal environments. In the montane warming treatment (MS), T_50_ and A_net_ were associated to growth, explaining 48% of its variability. T_50_ increased under warming with respect to the native temperature (**Figure 1**), presumably to minimise thermal damage in the hotter environment (Manzi et al., 2025). While A_net_ showed no significant change relative to native conditions, the associated T_optA_ increased significantly, enabling sustained high photosynthesis under elevated temperatures (**Figure 1**).

**Figure 3:**
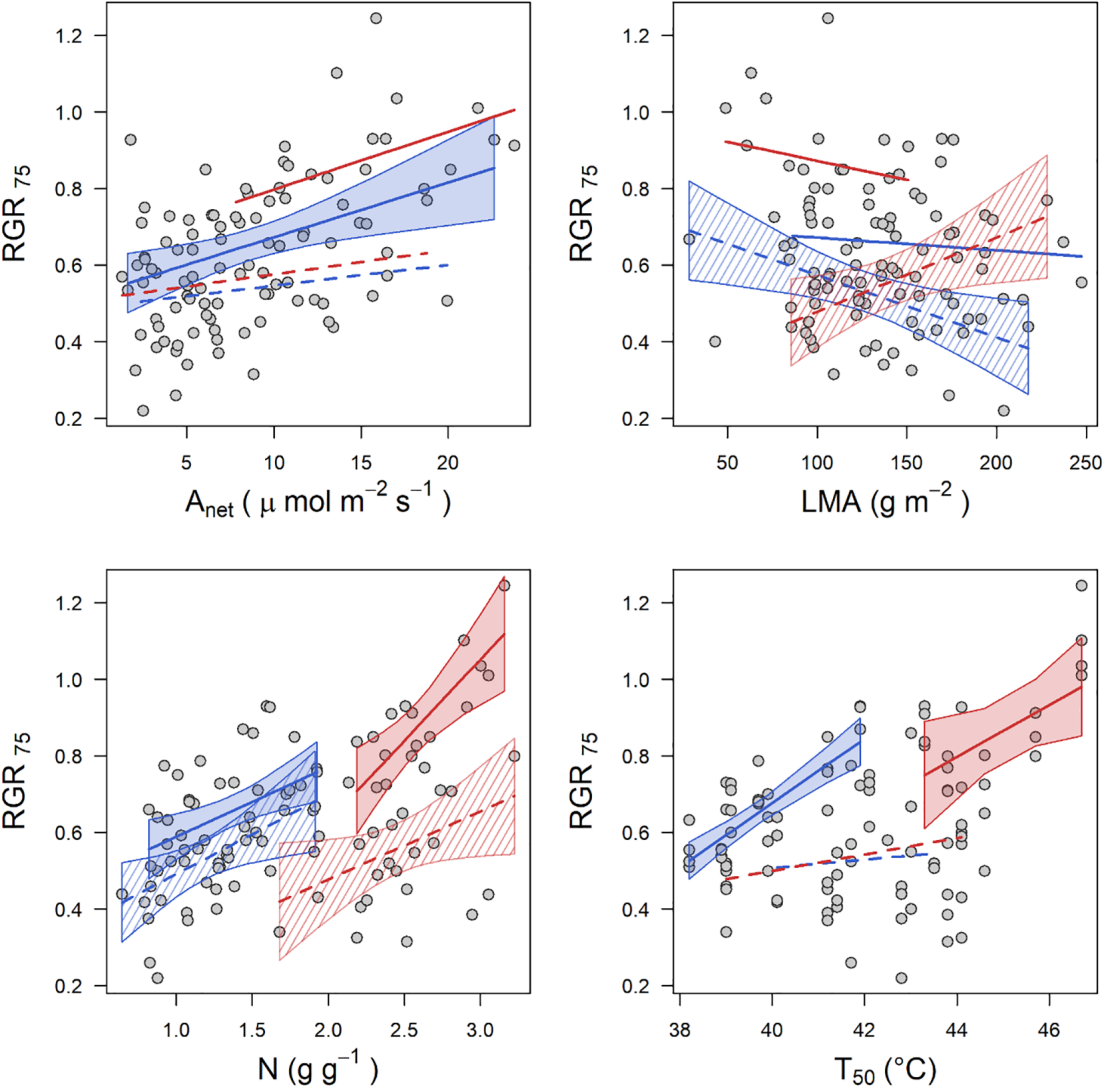
Relationship between the 75% relative growth rate (RGR_75_) and plant selected functional traits: **(a)** net photosynthetic rate (A_net_), **(b)** leaf mass per area (LMA), **(c)** leaf nitrogen content (N), and **(d)** thermotolerance threshold (T_50_). Red colour represents lowland species, while blue colour represents montane species. Solid lines and filled polygons indicate control conditions, whereas dashed lines and striped polygons indicate treatments (cooling for LS and warming for MS). Polygons represent 95% confidence intervals of linear models are only shown when the evaluated relationships are statistically significant.

Under lowland cooling, only two (N and T_optA_) of the six growth-associated traits (N, V_cmaxOpt_, T_optA_ T_50_, LMA, δ^13^C) lacked thermal plasticity (**Figure 3**). However, N which had a strong influence on RGR, was associated to A_net_ and V_cmaxOpt_ (**Supplementary Figure S1**), both of which significantly declined under cooling (**Figure 1**). Previous work examining photosynthetic temperature responses in the same species and experimental sites revealed relatively static responses in lowland species, despite significant declines in photosynthetic rates under cooling (Dusenge et al., 2025). LMA which had the strongest relationship on growth under LS cooling (**Figure 2**), increased significantly with respect to the native environment, consistent with a conservative strategy under stressful conditions (Poorter et al., 2009; Wright et al., 2003). In LS, high LMA may represent an adaptive shift enhancing structural function or efficiency under cooler temperatures (Poorter et al., 2009; Niinemets, 2015). In MS, however, high LMA may become maladaptive under warming, as denser leaves can incur higher construction and maintenance costs, potentially limiting net carbon gain. This conservative response in LS under cooling was observed in the same experiment in lowland species within six months of planting (Cox et al., 2024). Also δ^13^C increased under LS cooling indicating higher water use efficiency, aligning with the lower observed A_net_ and V_cmaxOpt_ (**Figure 1**) and consistent with adaptive responses noticed in the same lowland species that were observed within six months of planting (Cox et al., 2024). By focusing on juvenile trees, this study fills a gap in trait-based ecology, which has traditionally emphasised seedlings or mature individuals (Falster et al., 2010; Iida et al., 2014, but see Gibert et al., 2016). Growth during this developmental stage is a key predictor of long-term survival and recruitment, particularly under rapid environmental change. However, 52% of the variation in RGR_75_, particularly in MS under warming, remains unexplained (**Figure 2**). This suggests that unmeasured traits such as dark respiration, carbon allocation, or belowground architecture may play a role. For example, shifts in respiratory efficiency under warming could partially offset declines in photosynthetic performance, while changes in biomass allocation or root structure could influence access to limiting resources. Including these traits in future studies could enhance the explanatory power of trait–growth models and improve projections of species’ responses to climate change.

### Montane and lowland species differences across temperature treatments

Our SEM results highlight the expected relationship between photosynthetic traits and growth across both species groups and temperature treatments, supporting the established link between carbon assimilation and tissue formation (Shipley et al., 2006). While photosynthesis should theoretically translate into growth, environmental conditions modulate both photosynthetic efficiency and the extent to which it contributes directly to biomass accumulation (Fatichi et al., 2014; Körner, 2015). Additionally, thermotolerance also emerged as a key trait in both species’ groups and treatments, although its direction of association with growth varied—for instance, in LS under cooling, the relationship was opposite to that observed in other groups (**Figure 2**.). This pattern was supported by both individual trait models and SEM, where its predictive power was even stronger. These findings suggest potential interactions between thermotolerance and other leaf traits that ultimately affect growth. In essence, if leaves are protected from thermal damage, they may sustain higher photosynthetic rates, which in turn can lead to enhanced growth.

The emergence of water-use traits in the SEM models for LS species further highlights functional differences between species groups. LS species appear to be more water-demanding than MS species, which aligns with the ecological characteristics of the *Inga* genus, known for its high transpiration rates (Pennington and Fernandes, 1998). Because our common garden experiment was regularly irrigated to minimise water limitation and thereby isolate temperature effects as much as possible, LS species may have been favoured, enabling higher performance under control conditions and potentially mitigating the effects of thermal stress under cooling. This approach also reflects the relatively high mean annual precipitation of our Andean sites, which are generally well-watered environments. Nevertheless, the greater water requirements of LS species suggest they may be more vulnerable to drought than MS species—an expectation that warrants testing in future experiments.

Our results suggest that trait–growth relationships are not fixed but shift with environmental context. While photosynthetic traits (i.e. A_net_, V_cmaxopt_) were key drivers of growth under native conditions, structural traits (i.e. LMA) gained greater importance under thermal stress, most notably in LS under cooling. LS maintained growth in these conditions by relying on a diverse set of traits, including structural (i.e. LMA) and physiological traits (i.e. N, V_cmaxopt_) and water use (δ^13^C) traits, whereas thermotolerance traits (i.e. T_50_) showed weaker associations with growth. In contrast, although MS showed physiological variation in response to warming (Dusenge et al., 2025), the range of traits strongly associated with growth became more restricted. This suggests that while MS exhibit species-level variation that may reflect plastic responses, these may not fully compensate for warming-induced stress at the whole-plant level. This supports the view that LS may show greater functional effectiveness of their plasticity under new conditions, a potential driver for thermophilisation (Cox et al., 2024. In contrast, the growth performance of MS may be constrained not by the absence of plasticity but by the limitations in how that plasticity translates into sustained growth under elevated temperatures, increasing their vulnerability to warming and to competitive displacement by LS (Cox et al., 2024; Martínez-Villa et al., 2024).

We also found that whilst LMA is retained in all SEM models, it indirectly affected growth, except for LS under cooling. LMA is a trait simultaneously correlated to photosynthetic capacity, leaf lifespan and respiration that has been found to be a good proxy for tree growth (Shipley et al., 2006), highlighting its importance in providing an integrating signal with regard to tree functioning. High LMA is often associated with increased leaf tissue density and structural reinforcement, but also higher maintenance respiration costs (Poorter et al., 2009). One interpretation is that LS may experience higher respiratory costs under cooling conditions, therefore limiting carbon availability for growth. However, we did not directly measure respiration in this study. As such, this remains a possible explanation rather than a confirmed mechanism. Conversely, the negative LMA–growth relationship in MS under warming could reflect physiological stress, whereby high LMA leaves become inefficient in warm environments. Alternatively, the positive LMA–growth relationship in LS might indicate greater plasticity in leaf construction—i.e., LS may be able to maintain or enhance growth across a broader range of LMA values under cooling, potentially through compensatory mechanisms like increased nutrient-use efficiency or altered allocation patterns. While LMA proved to have strong associations to growth in novel temperature environments, its explanatory power was lower than that of the physiological traits observed under control conditions. This highlights that structural traits may become more influential when species experience environmental stress, but also that their effects are highly context-dependent, varying with species’ thermal origins and the direction of temperature change.

## Conclusion

This study provides clear evidence that functional leaf traits, particularly those related to photosynthesis and thermotolerance, and to a lesser extent, photosynthetic temperature responses, and structural characteristics, are key predictors of tropical tree growth across thermal gradients. We show that the strength and direction of trait–growth relationships are not fixed but shift depending on temperature context, with photosynthetic traits strongly associated to growth in native environments and T_50_ emerging as strong associations to growth under temperature change. This demonstrates that climate warming not only affects tree growth directly but also reshapes the trait–performance relationships that underpin forest productivity.

Our results highlight the need to incorporate temperature-sensitive traits into predictive models of forest response to climate change. By identifying specific traits that mediate growth under warming and cooling scenarios - important for uphill migrations in elevation gradients- we take an important step towards mechanistically linking trait data with ecosystem function in tropical montane forests. However, to develop a more complete mechanistic understanding, we recommend future studies quantify traits related to plant respiration, which were not assessed here but may be central to performance under elevated temperatures.

Overall, this work advances our understanding of how climate warming may restructure tropical montane forest communities, not only through changes in species composition but also via shifting trait–function relationships. This has direct implications for forecasting carbon cycle and biodiversity outcomes in these globally important ecosystems.

## Data Availability

The physiological data that support the findings of this study are openly available in Figshare at https://doi.org/10.6084/m9.figshare.29084276.v1. The tree growth data are available at NERC EDS Environmental Information Data Centre at https://doi.org/10.5285/c7ce1610-aba3-4a09-bf7c-1b6c774d597a.

## Supplementary material

Supplementary Figure 1 The initial path model (i.e. null hypothesis) used to predict 75% relative growth rate (RGR_75_, black box) from 19 leaf traits (See materials and methods) addition to species (15 species, black circle) and treatment (Control, Warming or Cooling, black circle) and correlations among traits (coloured rectangles). Black solid arrows show direct correlations tested. For the model starting point it was assumed that species group and temperature treatment could influence all traits, all traits could influence RGR_75_ and all traits could influence each other–that is, no initial assumptions were made about whether direct relationships were positive or negative. Colours indicate the groupings of plant traits as follows: green, photosynthetic; blue, water use; orange, temperature response; red, thermotolerance; purple, structural.

Supplementary Figure 2 Correlogram among all functional traits used in analyses for two species groups (Montane and Lowland) at the two temperature treatments (warming and cooling). Each panel represents a bivariate correlation between two traits indicated by row and column. Colours show the magnitude of Pearson R2 in which red colours are negative and blue colours positive correlation values. The polygon shape represents the data dispersion in each bivariate correlation. Plant traits outlined in the figure are defined in **Table 1**.
