# Supplementary material for: Linking leaf traits to growth responses under climate warming in tropical trees
Source: Front Plant Sci. 2025 Dec 2;16:1721483. doi: 10.3389/fpls.2025.1721483 (PMC12705590; doi:10.3389/fpls.2025.1721483)

Supplementary Figure 1 The initial path model (i.e. null hypothesis) used to predict 75 % relative growth rate (RGR_{75}, black box) from 19 leaf traits (See materials and methods) addition to species (15 species, black circle) and treatment (Control, Warming or Cooling, black circle) and correlations among traits (coloured rectangles). Black solid arrows show direct correlations tested. For the model starting point it was assumed that species group and temperature treatment could influence all traits, all traits could influence RGR_{75} and all traits could influence each other–that is, no initial assumptions were made about whether direct relationships were positive or negative. Colours indicate the groupings of plant traits as follows: green, photosynthetic; blue, water use; orange, temperature response; red, thermotolerance; purple, structural.

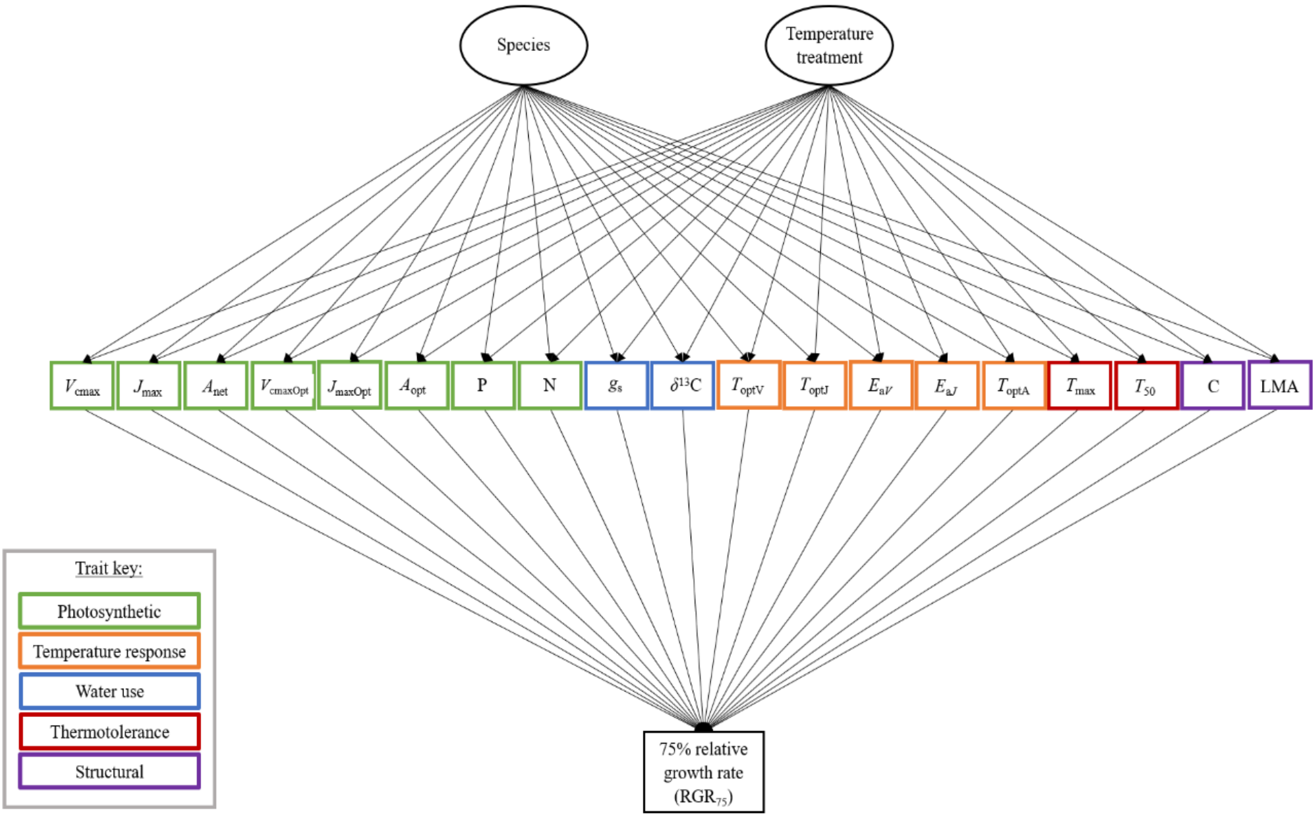

Supplement: Supplementary Figure 1 — The initial path model (i.e. null hypothesis) used to predict 75% relative growth rate (RGR75, black box) from 19 leaf traits (See materials and methods) addition to species (15 species, black circle) and treatment (Control, Warming or Cooling, black circle) and correlations among traits (coloured rectangles). Black solid arrows show direct correlations tested. For the model starting point it was assumed that species group and temperature treatment could influence all traits, all traits could influence RGR75 and all traits could influence each other–that is, no initial assumptions were made about whether direct relationships were positive or negative. Colours indicate the groupings of plant traits as follows: green, photosynthetic; blue, water use; orange, temperature response; red, thermotolerance; purple, structural. [file Image1.pdf]
