# Supplementary material for: Linking leaf traits to growth responses under climate warming in tropical trees
Source: Front Plant Sci. 2025 Dec 2;16:1721483. doi: 10.3389/fpls.2025.1721483 (PMC12705590; doi:10.3389/fpls.2025.1721483)

Supplementary Figure 2 Correlogram among all functional traits used in analyses for two species groups (Montane and Lowland) at the two temperature treatments (warming and cooling). Each panel represents a bivariate correlation between two traits indicated by row and column. Colours show the magnitude of Pearson R2 in which red colours are negative and blue colours positive correlation values. The polygon shape represents the data dispersion in each bivariate correlation. Plant traits outlined in the figure are defined in Table 1.

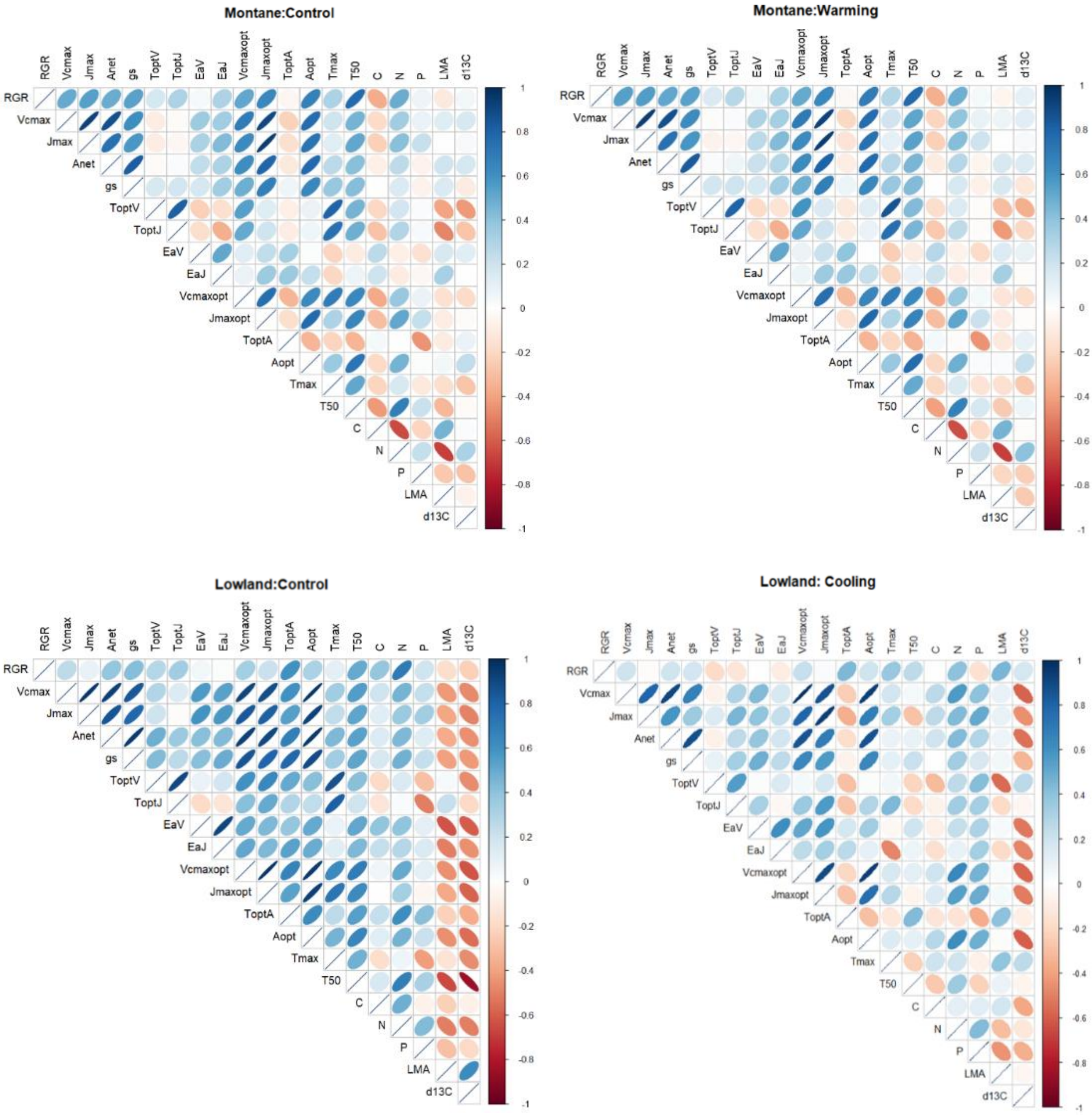

Supplement: Supplementary Figure 2 — Correlogram among all functional traits used in analyses for two species groups (Montane and Lowland) at the two temperature treatments (warming and cooling). Each panel represents a bivariate correlation between two traits indicated by row and column. Colours show the magnitude of Pearson R2 in which red colours are negative and blue colours positive correlation values. The polygon shape represents the data dispersion in each bivariate correlation. Plant traits outlined in the figure are defined in Table 1. [file Image2.pdf]
